# Interacting Quantum Atoms Analysis of Covalent and Collective Interactions in Single Elongated Carbon–Carbon Bonds

**DOI:** 10.3390/molecules30214316

**Published:** 2025-11-06

**Authors:** Antonio Bonesana-Espinoza, José Manuel Guevara-Vela, Evelio Francisco, Tomás Rocha-Rinza, Ángel Martín Pendás

**Affiliations:** 1 Instituto de Química, Universidad Nacional Autónoma de México, Circuito Exterior, Ciudad Universitaria, Delegación Coyoacán C.P., Mexico City 04510, Mexico; antoniojunioralbonesana@gmail.com; 2 School of Engineering and Physical Sciences, Heriot-Watt University, Edinburgh EH14 4AS, UK; j.guevara-vela@hw.ac.uk; 3 Departamento de Química Física y Analítica, Universidad de Oviedo, Av. Julián Clavería 8, 33006 Oviedo, Asturias, Spain; evelio@uniovi.es

**Keywords:** interacting quantum atoms, IQA, quantum theory of atoms in molecules, QTAIM, London dispersion, long carbon–carbon bonds, energy decomposition analysis

## Abstract

Chemical bonds among carbon atoms are central to chemistry. A general working principle regarding these interactions is that these contacts become stronger as the carbon atoms become closer to each other. Nevertheless, there are long, yet strong single C–C bonds that challenge this interpretation. Herein, we perform a quantitative thorough decomposition of the electronic energy of hexaphenylethane and several derivatives of this molecule with increasingly bulkier substituents. For this purpose, we exploit state-of-the-art methods of wave function analysis for the examination of the chemical bonding scenario in the examined systems, namely, the quantum theory of atoms in molecules (QTAIM) and the interacting quantum atoms (IQA) electronic energy partition. Our results reveal the predominance of collective non-covalent interactions over the central, covalent one in the chemical bonding of the examined molecules, in particular for those that have been synthesized in the laboratory. The QTAIM and IQA methods also showed that, besides London dispersion, electron sharing comprises an important contribution to the abovementioned collective interactions. Overall, our results give valuable insights about the importance of collective interactions in the investigated systems and they aid in the understanding of the nature of long, yet stable single C–C bonds.

## 1. Introduction

**Figure 1:**
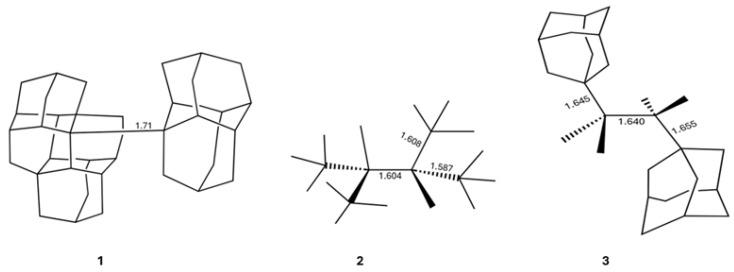
Illustrative hydrocarbons with long central C–C bonds that have been synthesized in the laboratory. The numbers indicate the lengths of the indicated chemical bonds in angstroms.

Bonds between carbon atoms lie at the heart of organic chemistry, underlying a variety of phenomena from the reactivity of small molecules to the architecture of advanced polymers. A basic tenet of chemical bonding holds that shorter bonds are typically stronger, reflecting greater orbital overlap and electron sharing, while long bonds indicate instability [1,2,3]. This correlation between bond length and bond strength underpins much of our chemical intuition and it is used constantly in the design of stable molecular frameworks [4,5]. However, a growing number of structurally characterized compounds appear to defy this expectation, displaying carbon–carbon single bonds that stretch well beyond conventional distances while remaining remarkably stable under ambient conditions [6,7,8]. Some of the most noticeable examples of this feature are diamondoid dimers, e.g., the molecule [121]tetramantane-diamantane **1** (Figure 1), which is a very stable molecule with melting point of 246 °C, and yet it has the longest C–C bond for an alkane up to now (1.71 Å) [9]. Another molecule having long, stable single C–C bonds is 1,1,2,2-tetra-tert-butyl ethane **2** (m.p. 150 °C) which presents three single C–C bonds wherein *d*(C–C) > 1.58 Å [10]. Likewise, 2,3-di-1-adamantyl-2,3-dimethyl butane **3** has three C–C bonds whose bond lengths are larger than 1.64 Å, and, still, the corresponding crystal has a melting point of 167 °C [11,12]. These anomalously long yet stable bonds challenge textbook models of covalent bonding and raise fundamental questions about what forces can sustain such elongated chemical bonds [12].

Among the forces capable of stabilizing weak interactions between atoms, London dispersion (LD), a long-range, non-directional interaction, plays a uniquely universal role. Arising from correlated fluctuations in the electron positions, LD acts as an attractive force on all matter, growing stronger with increasing molecular size and polarizability [13,14]. In systems with large, flexible substituents, this effect becomes particularly significant. What might appear at first glance as simple steric crowding can instead give rise to a network of favorable dispersion contacts that compensate for the energetic cost of bond elongation. Rather than destabilizing a stretched C–C bond, bulky groups may in some cases aid in its stabilization by collectively lowering the total energy of the system via LD [15]. A particularly conspicuous example is the effect of alkyl substituents on the hypothetical hexaphenylethane (HPE), (Ph)_3_C–C(Ph)_3_. Indeed, the introduction of large *tert*-butyl groups in all meta positions leads to the isolation of hexa(3,5-di-*tert*-butylphenyl) ethane which can be even characterized via X-ray crystal diffraction [16,17].

Experimental support for this view emerged most notably from the work of Schreiner and coworkers, who reported a series of remarkably stable alkanes with very long C–C bond lengths, as previously shown in Figure 1. These species, stabilized by bulky alkyl groups, are thought to owe their persistent long C–C single bonds largely to LD forces rather than conventional covalent bonding. This interpretation is based on computational studies, typically using dispersion-corrected density functional theory (e.g., PBE0-D3BJ) or high-level ab initio methods, such as CCSD(T), which suggest a central role for dispersion in the stabilization of these molecules. However, a detailed understanding of the energetic balance that makes such bonding possible remains incomplete. Disentangling the physical contributions that stabilize very long C–C bonds remains a central challenge. Many energy decomposition approaches, such as the local energy decomposition (LED) [18,19,20] or the symmetry-adapted perturbation theory (SAPT) [21,22,23], are highly informative for interfragment interactions, but they rely on perturbative treatments and an a priori fragmentation of the system [24]. In congested, covalently connected molecules, the strong density overlap across the C–C link violates the basic assumptions underlying these methods. As a result, such partitions may misattribute stabilization among electrostatics, exchange and dispersion, exhibit marked fragment-dependence, and yield trends that vary with the chosen scheme [24]. Together, these issues hinder quantitative comparison across a homologous series and they can lead to misleading design rules [25,26]. In contrast, real-space approaches within the field of quantum chemical topology (QCT) like the quantum theory of atoms in molecules (QTAIM) enable a fully non-perturbative, atom-centric view of bonding [27]. Among these, the interacting quantum atoms (IQA) method offers a rigorous decomposition of the total energy into chemically interpretable intra- and interatomic components [28,29]. IQA has been successfully applied to a wide range of problems, including chemical reactivity [30,31,32,33,34], the analysis of hydrogen bonding [35,36,37,38], non-covalent interactions [39,40,41,42,43], transition-metal complexes [44,45], and cooperative and anticooperative effects in non-covalent assemblies [46,47,48,49].

**Figure 2:**
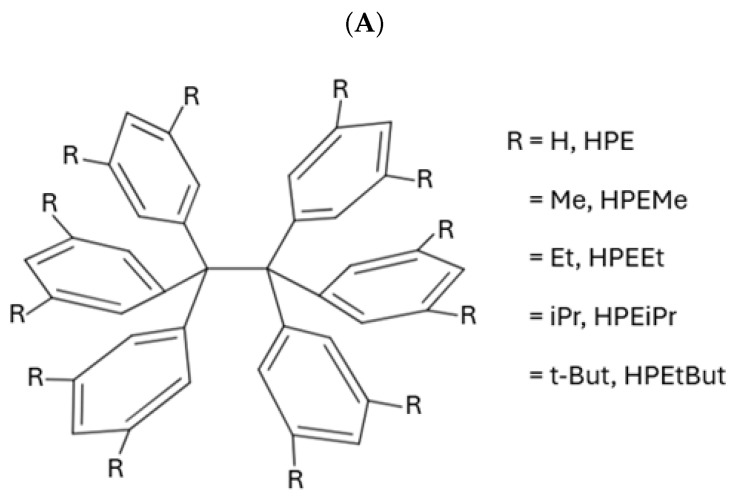

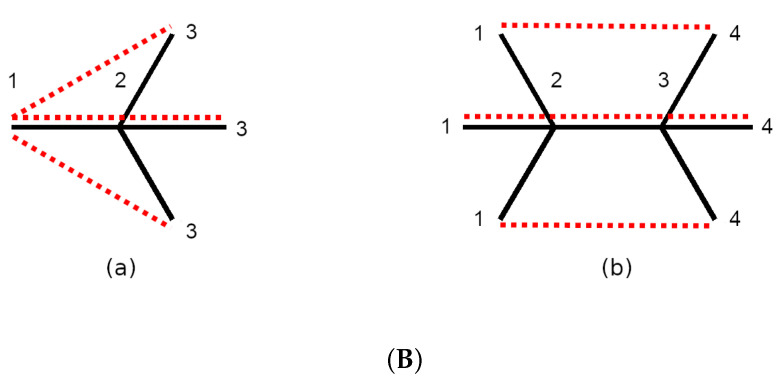
(**A**) Derivatives of hexaphenylethane addressed in this investigation. (**B**) Representation of collective interactions. (**a**) Collective 1–3 interactions: there exist standard covalent bonds associated with bond paths and bond critical points for interactions 1–2 and 2–3 indicated with solid black lines. Nevertheless, the system also presents relevant stabilizing collective 1–3 interactions represented with red dashed lines. (**b**) Collective 1–4 interactions: groups 1 and 4, which form standard covalent bonds with atoms 2 and 3, respectively, may present chemically stabilizing interactions as schematized with red dashed lines as well.

In this work, we apply QTAIM-based wave function analyses and the IQA partition of the electronic energy throughout a homologous series of compounds with long central C–C bonds to identify the most important energetic contributions in their stabilization. More specifically, we focused on the series of compounds shown in Figure 2A, i.e., a set of HPE derivatives with progressively bulkier substituents, allowing us to track interaction energies and to examine the chemical bonding scenario evolution across this set of compounds. Altogether, our results reveal that the QCT characterization of the strength of the central chemical C–C bond in the molecules shown in Figure 2A does not align with the computed dissociation energy of these systems. This observation indicates that there other factors that contribute to the stabilization of the molecule apart from the sole interaction between the carbon bonds in the ethane moiety. Indeed, the IQA analysis reveal the relevance of collective covalent 1–3 and, more importantly, 1–4 contacts (Figure 2B) in the compounds schematized in (Figure 2A) apart from LD. More broadly, these results highlight the essential features and the relevance of collective interactions and they aid in the understanding of both non-covalent interactions and covalent bonding in highly congested environments.

## 2. Results and Discussion

### 2.1. Potential Energy Curves and Thermodynamic Potentials

**Figure 3:**
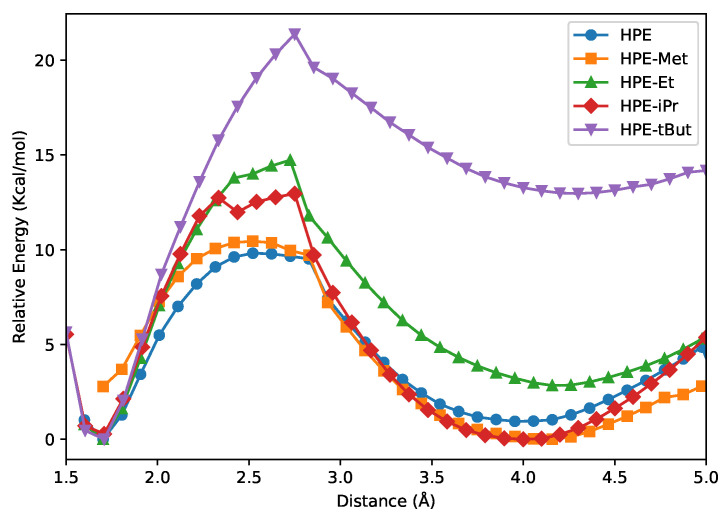
Potential energy curves corresponding to the relaxed scan along the central C–C distance of the molecules displayed in Figure 2A. In every point, only the C–C bond length is constrained, while all other geometry parameters are optimized. The energies are reported relative to the fully optimized minimum of the corresponding molecule.

Figure 3 reports the potential energy curves associated with the formation of the molecules schematized in Figure 2A from the corresponding free radicals. The associated enthalpy and Gibbs free energy of formation are reported in Table 1. We note that the aromatic groups severely impair the formation of the corresponding central C–C bond with respect to ethane. The potential energies curves reveal, as expected, the formation of a molecular complex which results from the interaction of the two free radicals followed by the occurrence of an energetic barrier preceding the formation of the central C–C bond. As can be noted from Table 1, the alkyl groups substituted on the phenyl groups of HPE increase the magnitude of the formation enthalpy of the central C–C bond, $\Delta H_{\mathrm{form}}$. This rise in $|\Delta H_{\mathrm{form}}|$ is consistent with the larger stability of HPEtBut with respect to HPE, as reflected in the feasibility of the synthesis of HPEtBut, as opposed to the elusiveness of the synthesis of HPE. Indeed, the formation of HPE from (Ph)_3_C· radicals is an endergonic process ($\Delta G_{\mathrm{form}}^{\mathrm{HPE}}=6.9$ kcal/mol), whereas the generation of HPEtBut from the corresponding (3,5,di-tertbutylC_6_H_3_)C· radicals is an exergonic reaction ($\Delta G_{\mathrm{form}}^{\mathrm{HPEtBut}}=-18.7$ kcal/mol) in the gas phase.

### 2.2. Quantum Theory of Atoms in Molecule Analysis

The above observations point to (i) a noticeable weakening of the C–C interaction due to the replacement of the hydrogens of ethane by phenyl groups, resulting in the molecules shown in Figure 2A and (ii) the strengthening of the central C–C bond as a result of the substitution with alkyl radicals of the meta position of the phenyl groups of HPE. Regarding the characterization of the chemical bonding scenario in these compounds, Table 2 reports selected topological properties of the electron density in the BCP associated with the central C–C bond of ethane along with those of the compounds presented in Figure 2A. The same chart displays the calculated bond lengths and formation energies of the corresponding systems. We note that the substitution of the ethane hydrogen atoms with aryl groups leads to a considerable reduction in the magnitude of the bond formation energy of the investigated central C–C bonds which equals up to 85% of $|\Delta E_{\mathrm{form}}|$. The corresponding changes in $\rho (r_{\mathrm{BCP}})$, δ(C,C) and $H(r_{\mathrm{BCP}})$ are much subtler, i.e., roughly 25%, 15%, and 40%, respectively. Certainly, the topological descriptors of ρ(r) of HPE, HPEMe, …HPEtBut are virtually the same, although the value of the formation energy of HPEtBut is twice as large as that of HPE. In other words, whilst the topological properties of ρ(r) are clearly different for (i) ethane on one hand, and (ii) the molecules shown in Figure 2A on the other, the same features of the topology of ρ(r) are very similar for HPE and its substituted analogues. Therefore, we proceeded to examine the chemical bonding scenario in these systems using other methods in the theoretical framework of QCT.

### 2.3. NCI Analyses

**Figure 4:**
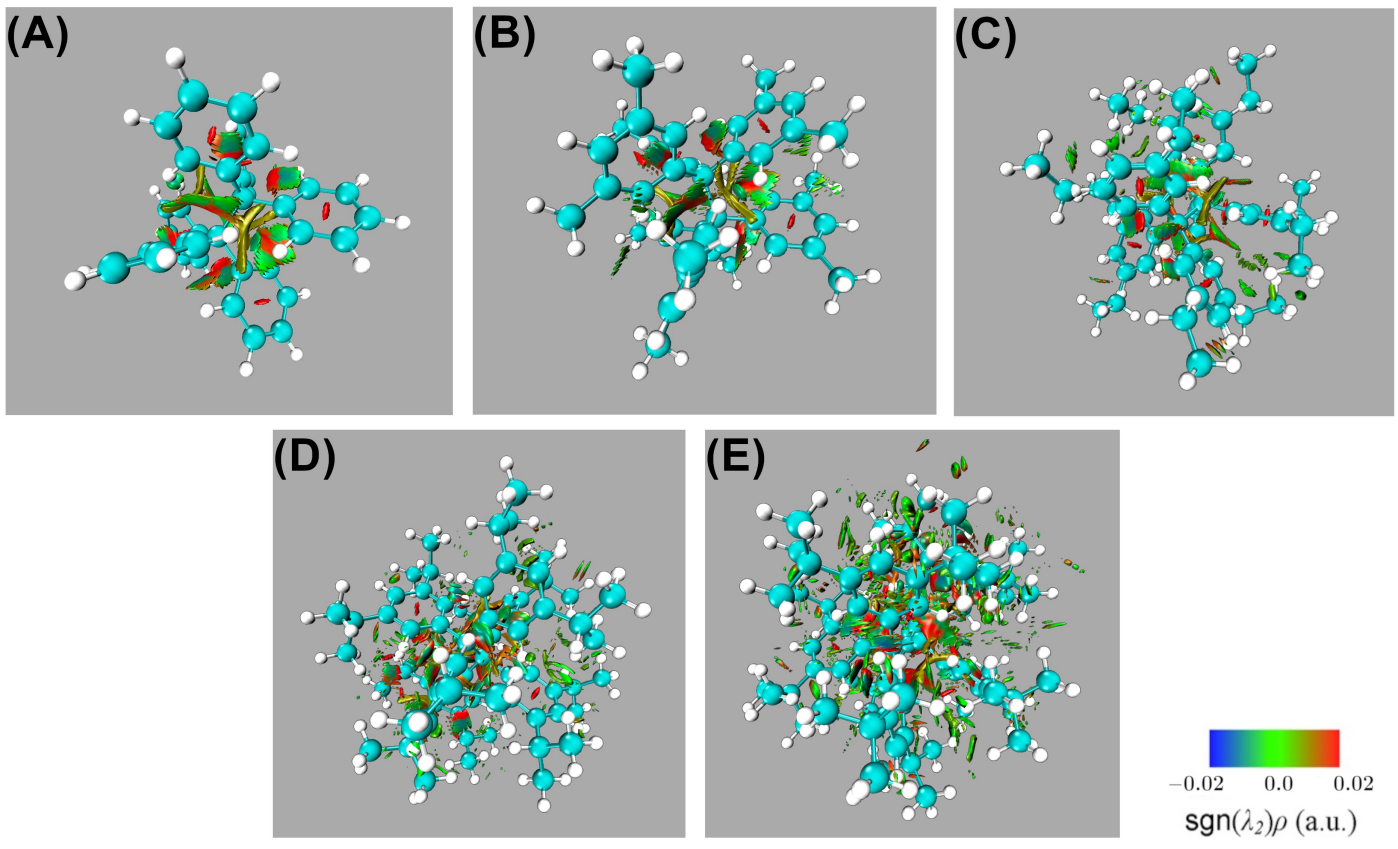
NCI index surfaces (s= 0.5 a.u.). We indicate the value of $\mathrm{sgn}\left(\lambda_{2}\right)\rho \left(r\right)$ via the scale in the bottom right part of the figure. The different panels correspond to (**A**) HPE, (**B**) HPEMe, (**C**) HPEEt, (**D**) HPEiPr and (**E**) HPEtBut.

Figure 4 shows NCI isosurfaces (s=0.5) colored by $\mathrm{sign}\left(\lambda_{2}\right)\rho \left(r\right)$. A clear monotonic trend appears across panels (A)–(E): as substituents grow, the number and extent of low-gradient regions increase, forming a dense network of contacts around the central C–C bond and among facing aryl/alkyl surfaces. The green lobes (weak van der Waals regime) percolate into quasi-continuous shells in the bulkiest systems, signaling extensive dispersion surfaces rather than isolated pairwise contacts. Red patches (repulsive) mark local steric clashes at short C–C non-bonded encounters. The overall picture supports that bulkiness amplifies the number of weak attractive contacts despite local repulsion, being consistent with a cooperative, many-contact stabilization that our IQA analysis attributes largely to exchange-correlation contributions, as discussed below.

### 2.4. Interacting Quantum Atoms Analyses

Regarding the division of the formation energy into deformation and interaction energies according to Equation (26), Table 3 reports the values of these quantities for the corresponding radicals involved in the formation of ethane and HPE. We performed this analysis on these two molecules because we could carry out the complete IQA analysis of these two species without any approximation. We note that although the IQA interaction energy between the Ph_3_C· radicals (−291.2 kcal/mol) is larger in magnitude than it is for the ·CH_3_ species (−196.3 kcal/mol), the very large deformation energy (300.9 kcal/mol) of the former system overwhelms its IQA interaction energy. This large deformation energy is related with the loss of planarity of the Ph_3_C· radicals along with the reduction of the π conjugated system of these radicals when the central C–C bond is formed in HPE. Indeed, the delocalization indices of the ipso carbons of the phenyl groups with the corresponding central carbon atom in hexaphenylethane are on average 0.950 a.u., whereas in the (Ph_3_)C· radical, the corresponding value is 1.130 a.u. In other words, there are roughly 0.180 fewer pairs of electrons shared between the central carbons with the ipso C due to the formation of the central C–C bond in HPE. Another relevant aspect of Table 3 is that the IQA interaction energy between the carbon atoms in ethane (−172.7 kcal/mol) is considerably larger than it is in HPE (−147.7 kcal/mol). The IQA interaction C–C energy in ethane represents roughly 88% of $E_{\mathrm{int}}^{H}3C\ldots {\mathrm{CH}}_{3}$, whereas in the case of hexaphenylethane it only amounts to 51% of $E_{\mathrm{int}}^{\mathrm{Ph}}3C\ldots {\mathrm{CPh}}_{3}$. In other words, the interaction energy of the central bond in HPE contributes substantially less to the stability of the system than it does in ethane. Therefore, there must be other interactions that contribute to the stability of HPE (the formation of HPE from two Ph_3_C· radicals is an exothermic process as reported in Table 1 and Table 2) which we examine below.

The abovementioned encounters are known as collective interactions which comprise a source of stabilization that does not arise from a single dominant pairwise contact; rather, these interactions emerge from the cooperative sum of many weak interatomic interactions distributed across fragments. Within the IQA framework, the magnitude of the collective interactions is computed as the sum of interatomic energies over all cross-fragment atom pairs excluding the central covalent bond. The magnitude of collective interactions typically increases with substituent size and contact area. Prototypes of this type of interaction were discussed for the ${\mathrm{NaBH}}_{3}^{-}$ and ${\mathrm{NaB}(\mathrm{CN})}_{3}^{-}$ anions, whose stability cannot be rationalized by any single Na⋯B or Na⋯H contact but, instead, by a network of many weak interactions; see the original proposal and subsequent debate for further details [50,51,52,53,54,55,56,57]. Figure 2B schematizes this notion, differentiating between 1–3 and 1–4 interactions.

### 2.5. Collective 1–3 Interactions

As abovementioned, the fact that the central C–C interaction in HPE is considerably less stabilizing than it is in ethane, and the formation of the former molecule from its corresponding radicals is still an exothermic process, is reminiscent of the concept of collective bonding. This notion refers to the existence of strong stabilizing chemical contacts that are associated with neither a chemical bond in the context of Lewis structures nor with a BCP as established by the QTAIM theoretical framework [50]. An important type of collective interaction is of the type 1–3, as shown in Figure 2(Ba). This is relevant in molecules such as ${\mathrm{NaBH}}_{3}^{-}$ and ${\mathrm{NaB}(\mathrm{CN})}_{3}^{-}$ wherein sodium establishes strong interactions with H atoms and with the CN functional groups, respectively. Furthermore, collective 1–3 interactions have been also described in organometallic systems such as organolithium and organomagnesium species [50]. Given this background, one can also examine the 1–3 collective interactions between one central carbon atom and the aromatic groups bonded to the other central carbon for the molecules shown in Figure 2A. Table 4 reports the approximate IQA interaction energy for collective 1–3 interactions in the molecules HPE, …, HPEtBut. We note that 1–3 contacts have relevant contributions to the overall stability of the molecules shown in Figure 2A. Nevertheless, the collective 1–3 interactions are very similar throughout the same series of molecules and therefore they are not the main reason for the larger stability of HPEtBut with respect to HPE. Hence, we decided to examine 1–4 interactions as depicted in Figure 2(Bb), videlicet among the aromatic groups bonded to the central atoms in the molecules in the series HPE, …, HPEtBut. The last-mentioned interactions seem to be, upon initial examination, similar to those existing among the fluorine atoms of $XF_{n}$ systems (*X: B* or *C*) [50]. These molecules present large F–F interatomic IQA exchange-correlation interactions throughout space [50]. This background made us hypothesize that 1–4 contacts are relevant in the chemical bonding of the examined systems as discussed below.

### 2.6. Collective 1–4 Interactions

Table 5 reports the IQA interaction energy corresponding to collective 1–4 interactions in HPE, that is to say, among the aromatic groups bonded to the central carbon atoms in the HPE molecule. This observation reveals the important role that these non-covalent interactions might have in the formation of the examined species. This situation occurs to the extent that the sum of the non-covalent interactions of the substituent phenyl groups, as reported in Table 5 (−115.928 kcal/mol), is comparable with that of the central C–C bond in HPE (−147.689 kcal/mol). Yet, another significant difference concerning ethane is the fact that the contribution from the dispersion energy is relevant in the formation HPE from the Ph_3_C· radicals. Without the dispersion energy component, the formation of HPE from these species would be computed as an endothermic process. Indeed, Table 3 indicates that without the dispersion correction, the formation energy of HPE from two ·C(Ph_3_) radicals is 9.637 kcal/mol, whereas the contribution of LD adds up to −20.884 kcal/mol, leading to an exothermic formation energy of −11.246 kcal/mol. Still, the exothermic character of the interaction of two ·C(Ph_3_) is overcome by the entropy decrease that accompanies the formation of a chemical bond in the gas phase.

Concerning the other compounds in Figure 2A, Table 6 displays the approximate IQA interaction energies for the molecules shown in Figure 2A (Equations (16)–(18)). We note that the IQA interaction energies among the phenyl groups increase with the bulkiness of the alkyl substituents on the phenyl groups, whereas the corresponding quantity for the central carbons $E_{\mathrm{int}}^{C\cdots C}$ in the molecules HPE, …HPEtBut remains virtually constant. The larger increase in such IQA interaction energies occurs in the change from HPEiPr (−107.0 kcal/mol) to HPEtBut (−129.8 kcal/mol), i.e., $|\Delta E_{\mathrm{int}}^{\phi \cdots \phi }|=22.8\;\mathrm{kcal}/\mathrm{mol}$. This situation results in a larger IQA interaction energy among the aromatic groups than for the central carbon atoms in HPEEt (−99.0 with respect to −85.2 kcal/mol), HPEiPr (−107.0 with respect to −85.1 kcal/mol), and HPEtBut (−129.8 with respect to −86.0 kcal/mol). Another important issue is that the IQA interaction energy among the phenyl groups greatly surpasses the contributions to dispersion in the five examined molecules: HPE (−69.9 with respect to −16.7 kcal/mol), HPEMe (−78.0 with respect to −23.0 kcal/mol), HPEEt (−99.0 with respect to −29.1 kcal/mol), HPEiPr (−107.0 with respect to −33.8 kcal/mol), and HPEtBut (−129.8 with respect to −42.1 kcal/mol). This comparison indicates that other effects apart from LD, e.g., through-space electron sharing, might be important in the chemical bonding of these molecules. Given the importance of the IQA interaction energy among the phenyl groups bonded to the central carbon atoms, we consider the division of the IQA interaction energy into their classical and exchange-correlation contributions according to Equation (16). Because of the similar electronegativities of carbon and hydrogen, the IQA classical component among the phenyl groups bonded to the central carbon atoms is negligible (≈0.2 kcal/mol); therefore, the exchange-correlation component is dominant among these groups. This observation is relevant because of the suggestion that single elongated C–C bonds are stabilized via LD. The data in Table 6 indicates that the exchange-correlation contribution among the phenyl groups in the examined molecules considerably outweighs the dispersion component. We do not imply with this statement that LD is irrelevant for the description of the chemical bonding within the investigated molecules. For example, we mentioned above that without LD the formation of HPE from the ·C(Ph_3_) free radicals would have been calculated as an endothermic process. Nevertheless, we emphasize that the covalent contributions are also significant in the interaction of the aromatic groups of HPE,…, HPEtBut. The relevance of covalency among the aromatic groups bonded to the central carbon atoms of the molecules shown in Figure 2A is evidenced by the delocalization indices among these groups, as shown in Table 7. The values of δ(C,C) for the central carbon–carbon bond in the series HPE, …, HPEtBut are roughly ≈0.84–0.86 atom units. We note that the sum of the delocalization indices between the aromatic rings is, indeed, greater than that between the central C–C atoms. This circumstance occurs to the extent that the numbers of delocalized pairs of electrons among individual aromatic rings 1–4 (0.593), 2–5 (0.594), and 3–6 (0.574) for HPEtBut in Table 7 are comparable with the corresponding value for the central C–C bond (0.865). A similar situation occurs for the exchange-correlation energy among the abovementioned pairs of aromatic rings at the bottom of Table 6. The IQA exchange-correlation among the same aromatic rings represents circa 40% of the corresponding value for the central C–C bond in this system. These data support the relevance of electron-sharing among the aromatic rings in the chemical bonding of the molecules schematized in Figure 2A, in particular, of HPEtBut, as well as the relevance of collective 1–4 interactions within this system.

### 2.7. Comparison Among Covalent 1–2 Versus Collective 1–3 and 1–4 Interactions

We proceed now to compare the relevance of the traditionally covalent 1–2 interactions with respect to the collective 1–3 and 1–4 interactions in the formation of the series HPE, …, HPEtBut from its corresponding free radicals. The data reported in Table 4 and Table 6 reveal that non-covalent interactions are relevant in the formation of these molecules via the following process:

(1) $$2{\left(R_{2}\phi \right)}_{3}C\cdot ⇌{\left(R_{2}\phi \right)}_{3}C-C{\left(R_{2}\phi \right)}_{3}.$$

In order to quantitatively assess the relevance of non-covalent, collective 1–3 and 1–4 interactions over those of covalent 1–2 interactions, we define the index $\Theta_{\mathrm{col}/\mathrm{cov}}$ as

(2) $$\Theta_{\mathrm{col}/\mathrm{cov}}=\frac{E_{\mathrm{col}}^{1-3}+E_{\mathrm{col}}^{1-4}}{E_{\mathrm{cov}}^{1-2}}.$$

The smaller the value of $\Theta_{\mathrm{col}/\mathrm{cov}}$, the more “conventional”, i.e., covalently-dominated, the interaction between the free radicals in Equation (1). The larger the value of $\Theta_{\mathrm{col}/\mathrm{cov}}$, the more the interaction between these radicals is dominated by collective interactions. Table 8 reports the values of $\Theta_{\mathrm{col}/\mathrm{cov}}$ for the molecules examined throughout this investigation. We note that whilst $\Theta_{\mathrm{col}/\mathrm{cov}}$ is fairly small for ethane (0.141), it is larger than unity for the molecules HPE, …, HPEtBut. This result means that collective contacts are more important than the central covalent C–C bond in the interaction of the ${\left(R_{2}\phi \right)}_{3}C\cdot$ species in the chemical Equation (1). This situation occurs to the point that $\Theta_{\mathrm{col}/\mathrm{cov}}=2.288$ for HPEtBu. If we consider the total of covalent and collective contacts in the interaction of the free radicals that ultimately comprise HPEtBu, the collective interactions represent circa 70% of the total IQA interaction energy among the two free radicals. The values reported in Table 4 and Table 6 imply that 1–4 collective contacts are more important than their 1–3 counterparts. Nevertheless, we note that the total contribution of the energy associated with 1–3 encounters is comparable to the dispersion interactions reported in Table 6. Furthermore, the approximations to the IQA interaction energy in Equations (16)–(18) underestimate the magnitude of $E_{\mathrm{int}}^{\mathrm{AB}}$ as evidenced by comparing the values reported in Table 5 for HPE and in the top part of Table 6 concerning the interaction of the phenyl groups in opposite sides of the central C–C in this molecule. These observations indicate that in spite of the fact that the collective 1–3 interactions do not represent the dominant factor in the bonding of the investigated molecules, they represent a significant contribution similar to the dispersion of the aromatic moieties considered in Table 6; therefore they should be taken into consideration in the formation of the investigated molecules from their corresponding radicals.

## 3. Theoretical Framework

The field of QCT comprises a series of methods of wave function analysis based on the topological study of distinct scalar fields derived from the electronic state vector. The origin of QCT resides on the QTAIM which relies on the topographical examination of the electron density, ρ(r). In the following lines, we show that the charge distribution equals the expectation value of the Dirac observable $\sum_{i=1}^{N}\delta \left(r_{i}-r\right)$, i.e.,

(3) $$\rho \left(r\right)=\langle \sum_{i=1}^{N}\delta \left(r_{i}-r\right)\rangle ,$$

wherein r is the position vector and $r_{i}$ is the position of the *i*-th electron in an *N*-electron system. Because of the indistinguishability of the electrons, one may write

(4) $$\begin{array}{cc} \langle \sum_{i=1}^{N}\delta \left(r_{i}-r\right)\rangle & =\overset{N\;\mathrm{times}}{\overset{︷}{\int \ldots \int }}\Psi^{★}\left(x_{1},\ldots ,x_{N}\right)\sum_{i=1}^{N}\delta \left(r_{i}-r\right)\Psi \left(x_{1},\ldots ,x_{N}\right)dx_{1}\ldots dx_{N}\\ \; & =N\overset{N\;\mathrm{times}}{\overset{︷}{\int \ldots \int }}\Psi^{★}\left(x_{1},\ldots ,x_{N}\right)\delta \left(r_{1}-r\right)\Psi \left(x_{1},\ldots ,x_{N}\right)dx_{1}\ldots dx_{N}, \end{array}$$

wherein $x_{i}=\left(r_{i},\omega_{i}\right)$ denotes the position and spin coordinates of the *i*-th electron. By integrating the position coordinate of electron 1 in the previous expression, we obtain

(5) $$\langle \sum_{i=1}^{N}\delta \left(r_{i}-r\right)\rangle =N\overset{N\;\mathrm{times}}{\overset{︷}{\int \ldots \int }}{\left|\Psi \left(r,\omega_{1},x_{2},\ldots ,x_{N}\right)\right|}^{2}d\omega_{1}dx_{2}dx_{3}\ldots dx_{N},$$

in which we exploit the properties of the Dirac delta function. Finally, by changing the order of integration in the former equation, we have

(6) $$\begin{array}{cc} \langle \sum_{i=1}^{N}\delta \left(r_{i}-r\right)\rangle & =\int \left(N\overset{N-1\;\mathrm{times}}{\overset{︷}{\int \ldots \int }}{\left|\Psi \left(r,\omega_{1},x_{2},\ldots ,x_{N}\right)\right|}^{2}dx_{2}dx_{3}\ldots dx_{N}\right)d\omega_{1}\\ \; & =\int \rho \left(r,\omega_{1}\right)d\omega_{1}=\rho \left(r\right), \end{array}$$

wherein $\rho (r,\omega_{1})$ is the spin-dependent electron density. Because the electron density is completely given by the state vector, the QTAIM analysis is orbital-invariant and independent on elements of the particular model of computation (e.g., basis sets). The QTAIM involves the determination of the critical points of ρ(r), i.e., points in which ∇ρ(r) vanishes [58]. Typically, molecular stable structures present critical points with range r=3, that is to say, with three curvatures different from zero [58]. These curvatures equal the eigenvalues of the Hessian of ρ(r) evaluated at the corresponding critical point $r_{c}$, i.e.,

(7) $$H\left(\rho \left(r_{c}\right)\right)=\left(\begin{array}{ccc} \frac{\partial^{2}\rho \left(r_{c}\right)}{\partial x^{2}} & \frac{\partial^{2}\rho \left(r_{c}\right)}{\partial x\partial y} & \frac{\partial^{2}\rho \left(r_{c}\right)}{\partial x\partial z}\\ \frac{\partial^{2}\rho \left(r_{c}\right)}{\partial y\partial x} & \frac{\partial^{2}\rho \left(r_{c}\right)}{\partial y^{2}} & \frac{\partial^{2}\rho \left(r_{c}\right)}{\partial y\partial z}\\ \frac{\partial^{2}\rho \left(r_{c}\right)}{\partial z\partial x} & \frac{\partial^{2}\rho \left(r_{c}\right)}{\partial z\partial y} & \frac{\partial^{2}\rho \left(r_{c}\right)}{\partial z^{2}} \end{array}\right).$$

Besides the rank, critical points of ρ(r) are characterized according to their signature, *s*, which equals the algebraic sum of the signs of the curvatures at the critical [58] point. In other words, critical points can be associated with the ordinate pair (r,s). The critical points with r=3 are related with different elements of molecular structure. Namely, the critical points with r=3, i.e., (3,−3), (3,−1), (3,+1), and (3,+3), indicate the occurrence of nuclei, chemical bonds between two atoms, rings, and cages, correspondingly. Hence, these critical points are, respectively, denoted as nuclear critical points (NCPs), bond critical points (BCPs), ring critical points (RCPs), and cage critical points (CCPs) [58].

Indeed, the chemical bond between two atoms can be characterized via the values of ρ(r) as well as of other scalar fields at the corresponding BCP. These scalar fields include, for example, ρ(r), $\nabla^{2}\rho \left(r\right)$, and the density of energy H(r) [27,58,59].

By considering the dynamical system established by ∇ρ(r), the QTAIM defines a division of the 3D space in atomic basins, A, B, …which are related with the atoms of chemistry [58]. An atomic basin is defined as the stable manifold of an NCP. The atomic basins in QTAIM are separated by an interatomic surface, i.e., the stable manifold of a BCP, which satisfies the zero-flux condition

(8) $$\nabla \rho \left(r\right)\cdot \hat{n}\left(r\right)=0,$$

wherein $\hat{n}\left(r\right)$ is a normal vector to the interatomic surface. Because QTAIM atomic basins are proper open quantum subsystems, one can calculate atomic expectation values of quantum mechanical operators. For example, the average number of electrons within an atom A is

(9) $$N_{A}=\int_{A}\rho \left(r\right)dr,$$

and the corresponding QTAIM charge is

(10) $$Q^{A}=Z_{A}-N_{A}.$$

in which $Z_{A}$ is the atomic number of the nucleus inside basin A. The QTAIM also defines the delocalization index between two atoms A and B, δ(A,B), as

(11) $$\delta \left(A,B\right)=-2\mathrm{cov}\left(N_{A},N_{B}\right),$$

in which cov(x,y) is the covariance of the two random variables *x* and *y* and $N_{A}$ is defined in Equation (9). The value of δ(A,B) is the number of electrons shared by the basins A and B; therefore, it is a measure of the relevance of covalency in the interaction of these two QTAIM atoms.

Regarding the IQA method for partitioning the electronic energy, we point out that this approach is based on the calculation of the electronic energy in terms of the first-order reduced density matrix $\rho_{1}\left(r_{1},r_{1}^{\;\prime }\right)$ and the pair density $\rho_{2}\left(r_{1},r_{2}\right)$ [60]

(12) $$E=\int_{r_{1}^{\;\prime }=r_{1}}\hat{h}\left(r_{1}\right)\rho_{1}\left(r_{1},r_{1}^{\;\prime }\right)dr_{1}+\frac{1}{2}\int \int \frac{\rho_{2}\left(r_{1},r_{2}\right)}{r_{12}}dr_{1}dr_{2}+V_{\mathrm{nn}},$$

in which $\hat{h}$ denotes the monoelectronic part of the electronic Hamiltonian,

(13) $$\hat{h}\left(r_{1}\right)=-\frac{1}{2}\nabla_{1}^{2}-\sum_{A}\frac{Z_{A}}{r_{A1}},$$

and $V_{\mathrm{nn}}$ is the nuclear repulsion. After establishing a partition of the 3D space into atomic basins, as QTAIM provides, one can divide the integrals in Equation (12) in regions corresponding to the atomic basins, and, hence, one may partition the electronic energy in different real-space contributions. By collecting the terms that depend upon particles on (i) one atomic and (ii) two atomic basins, one can define intra- ($E_{\mathrm{net}}^{A}$) and interatomic energies, respectively, ($E_{\mathrm{int}}^{\mathrm{AB}}$) [28,29,61] which fulfill the following condition:

(14) $$E=\sum_{A}E_{\mathrm{net}}^{A}+\frac{1}{2}\sum_{A\neq B}E_{\mathrm{int}}^{\mathrm{AB}},$$

As established in Equation (12), the contributions to $E_{\mathrm{net}}^{A}$ and $E_{\mathrm{int}}^{\mathrm{AB}}$ can be computed completely in terms of $\rho_{1}\left(r_{1},r_{1}^{\;\prime }\right)$ and $\rho_{2}\left(r_{1},r_{2}\right)$. Although Kohn–Sham DFT does not define any of these scalar fields, it is possible to introduce very reasonable approximations that allow an IQA partition energy based on the Kohn–Sham orbitals. By virtue of the partition of the pair density in a Coulombic and exchange-correlation term [60],

(15) $$\begin{array}{cc} \rho_{2}\left(r_{1},r_{2}\right) & =\rho \left(r_{1}\right)\rho \left(r_{2}\right)+\rho_{2}^{\mathrm{xc}}\left(r_{1},r_{2}\right)\\ \; & =\rho_{2}^{J}\left(r_{1},r_{2}\right)+\rho_{2}^{\mathrm{xc}}\left(r_{1},r_{2}\right), \end{array}$$

the IQA interaction energy between atoms A and B, $E_{\mathrm{int}}^{\mathrm{AB}}$, can be split into classical ($E_{\mathrm{cl}}^{\mathrm{AB}}$) and exchange-correlation components, ($E_{\mathrm{xc}}^{\mathrm{AB}}$)

(16) $$E_{\mathrm{int}}^{\mathrm{AB}}=E_{\mathrm{cl}}^{\mathrm{AB}}+E_{\mathrm{xc}}^{\mathrm{AB}},$$

which are, respectively, associated with the ionic and the covalent contributions of the interaction between atoms A and B. The consideration of the leading terms of the multipole expansion of $E_{\mathrm{cl}}^{\mathrm{AB}}$ and $E_{\mathrm{xc}}^{\mathrm{AB}}$ permits to approximate these two quantities as

(17) $$\begin{array}{cc} E_{\mathrm{cl}}^{\mathrm{AB}} & \approx TQ^{A}Q^{B}+T_{\alpha }\left(Q^{A}\mu_{\alpha }^{B}-Q^{B}\mu_{\alpha }^{A}\right)-T_{\alpha \beta }\mu_{\alpha }^{A}\mu_{\beta }^{B}, \end{array}$$

(18) $$\begin{array}{cc} E_{\mathrm{xc}}^{\mathrm{AB}} & \approx -T\frac{\delta (A,B)}{2}. \end{array}$$

wherein the Greek letters α and β denote Cartesian components, $\mu_{\alpha }^{X}$ is the α component of the dipole vector $\mu^{X}$, and

(19) $$\begin{array}{cc} T & =\frac{1}{R}, \end{array}$$

(20) $$\begin{array}{cc} T_{\alpha } & =-\frac{R_{\alpha }}{R^{3}}, \end{array}$$

(21) $$\begin{array}{cc} T_{\alpha \beta } & =\frac{3R_{\alpha }R_{\beta }-\delta_{\alpha \beta }R^{2}}{R^{5}}. \end{array}$$

The IQA partition energy allows the gathering of atoms in functional groups, G,H,I…whose net and interaction energies are given by

(22) $$\begin{array}{cc} E_{\mathrm{net}}^{G} & =\sum_{A\in G}E_{\mathrm{net}}^{A}+\frac{1}{2}\sum_{A\neq B}E_{\mathrm{int}}^{\mathrm{AB}}, \end{array}$$

(23) $$\begin{array}{cc} E_{\mathrm{int}}^{G\;H} & =\sum_{\begin{array}{c} A\in G,\\ B\in H \end{array}}E_{\mathrm{int}}^{\mathrm{AB}}, \end{array}$$

respectively. A similar equation to expression (16) holds for $E_{\mathrm{cl}}^{G\;H}$ and $E_{\mathrm{xc}}^{G\;H}$, so that

(24) $$E_{\mathrm{int}}^{G\;H}=E_{\mathrm{cl}}^{G\;H}+E_{\mathrm{xc}}^{G\;H}.$$

Finally, the energy associated with the formation of a G⋯H adduct, $\Delta E_{\mathrm{form}}$,

(25) $$G+H⇋G\cdots H,\;\Delta E_{\mathrm{form}},$$

is given by

(26) $$\Delta E_{\mathrm{form}}=E_{\mathrm{def}}^{G}+E_{\mathrm{def}}^{H}+E_{\mathrm{int}}^{G\;H},$$

wherein $E_{\mathrm{def}}^{I}$ (I=G,H) is the deformation energy of I within the adduct G⋯H, i.e., the difference in energy of I in (i) the adduct G⋯H and in (ii) its isolated, equilibrium configuration.

Finally, another tool of QCT that we exploit in this investigation is the non-covalent interaction (NCI) index. This index provides a simple real-space scalar field to locate and visualize weak interactions by analyzing regions of low electron density and low density gradient [62]. The NCI-index is based on the reduced density gradient

(27) $$\begin{array}{c} s\left(r\right)=\frac{1}{2{\left(3\pi^{2}\right)}^{1/3}}\frac{\left|\nabla \rho (r)\right|}{\rho {\left(r\right)}^{4/3}}, \end{array}$$

whose small values highlight spatial domains where non-covalent contacts occur. To distinguish the nature of these contacts, the NCI maps are colored using $\mathrm{sign}(\lambda_{2})\;\rho$ over isosurfaces of the reduced density gradient, where $\lambda_{2}$ is the second eigenvalue of the Hessian of ρ(r). Negative values of $\lambda_{2}$ (usually portrayed with blue) indicate attractive interactions such as hydrogen bonding or dispersion-dominated contacts, values near zero (normally displayed with green) correspond to weak van der Waals regimes, and positive $\lambda_{2}$ values (commonly shown in red) signal steric repulsion. Plotting isosurfaces of s(r) at a low threshold, colored by $\mathrm{sign}(\lambda_{2})\;\rho$, thus, yields an intuitive picture of where weak interactions stabilize or destabilize an electronic system. NCI does not provide energies by itself, but it complements QTAIM and IQA by revealing the spatial extent and character of the interactions that those methods quantify.

## 4. Computational Details

All molecular structures were optimized with ORCA (version 6) [63] using the PBE0 functional [64] with D3 dispersion including Becke–Johnson damping [65,66,67] and the def2-SVP basis set [68]. All Gibbs free energies and enthalpies are reported at 298.15 K and 1 atm. Entropies and thermal corrections were obtained from analytic harmonic frequencies at the same level of theory, ideal gas, rigid rotor, harmonic oscillator treatment (including translational, rotational, and vibrational contributions). No frequency scaling, quasi-harmonic, or hindered-rotor corrections were applied. All reported structures were confirmed as true minima (no imaginary frequencies). We chose the PBE0-D3BJ functional after we had considered the formation of HPE from the triphenylmethyl radical, (Ph_3_)C·,

(28) $$2{\left(\mathrm{Ph}\right)}_{3}C\cdot ⇋{\left(\mathrm{Ph}\right)}_{3}{C\text{-}C(\mathrm{Ph})}_{3},$$

with several exchange-correlation functionals and basis sets (Table S1). We considered as benchmark the results of the DLPNO-CCSD(T)/cc-pVTZ approximation which describes the formation of HPE from two (Ph_3_)C· radicals as an exothermic reaction. We note that apart from M06-2X, those functionals without dispersion corrections predict reaction (28) to be endothermic, in concordance with previous studies addressing single, long C–C bonds [12,69,70,71]. Furthermore, the methods M06/Def2-SVP and PBE0+D3BJ/Def2-SVP are the approximations that approach most closely the results of the benchmark calculations. Nevertheless, the M06-2X functional may produce electron densities that may present some instabilities [72] which can make them unsuitable for QTAIM analysis; hence, we chose the PBE0+D3BJ/Def2-SVP approximation to described the systems herein. We did not consider the Def2-TZVP approximation due to its strict computational requirements to address the largest systems considered in this work. The resolution-of-the-identity approximation [73,74] was employed for the Coulomb term and the chain-of-spheres (COSX) approximation [75,76] for the exchange-correlation one with the corresponding def2/J auxiliary basis [77]. Thermochemical corrections were obtained from analytical harmonic frequency calculations at the same level of theory. QTAIM analyses were performed with AIMAll (version 19.10.12) [78]; full IQA partitions and delocalization indices were computed for ethane and hexaphenylethane, whereas for the remaining derivatives we employed the first-order approximation to the IQA interatomic energies. We acknowledge that the PBE0+D3BJ geometry is not optimal for the computation of the PBE0 electron density. Nevertheless, dispersion corrections are necessary for the proper description of the energetics of the systems and processes investigated herein [12,69,70,71] In addition, the PBE0 and PBE + D3BJ structures are very similar (RMSD = 0.06 Å). We also point out that we also took into account these dispersion corrections throughout the decomposition of the electronic energies addressed herein. Non-covalent interaction (NCI) analysis was carried out with the Multiwfn [79,80] package, and the isosurfaces were rendered with VMD [81]. Finally, we computed interaction collectivity indexes (expression (2) vide infra), defined in terms of the sum of the IQA interatomic energies (Equation (23)), for the 1–3 and 1–4 cross-fragment contacts. This metric provides a compact measure of how much stabilization arises from many weak, non-bonded contacts rather than from the central C–C pair and it is inspired by the interaction collectivity index, ICI [50].

## 5. Conclusions

We presented herein a thorough examination of the chemical bonding scenario of HPE and its analogues substituted with methyl, ethyl, isopropyl, and tert-butyl in meta position with respect to the ipso carbon of the aromatic groups in these compounds. We found that the central C–C bond in this series of molecules is severely weakened as a result of the substitution of aromatic groups with respect to ethane. This weakening of the central C–C bond is ameliorated by non-covalent collective 1–3 and 1–4 interactions, the latter being considerably larger in magnitude. The collective 1–4 contacts are so important that they overwhelm the interaction energy of the central C–C bonds in the examined systems. The most conspicuous example of this statement is HPEtBu, for which collective interactions represent circa 70% of the interaction among the corresponding free radicals forming this molecule, as revealed by the analysis of the index $\Theta_{\mathrm{col}/\mathrm{cov}}$. Our results also evidence the importance of London dispersion in the chemical bonding of the examined species. Nevertheless, the QTAIM and IQA methods of wave function analysis reveal that electron sharing represents the most important contribution to the energetics of 1–4 collective contacts; therefore, this component should be considered along with LD in the interpretation of the stability of the examined compounds. Altogether, the results of this investigation highlight the importance and nature of non-collective interactions in the investigated systems and they aid in the understanding of the essential features of long, yet stable, single C–C bonds.

## Figures and Tables

**Table 1:** Enthalpy and Gibbs free energy of formation for ethane and the molecules schematized in Figure 2A from the corresponding free radicals whose interaction results in the central C–C bond of the molecule.

Molecule	$\Delta H_{\mathrm{form}}$/kcal·${\mathrm{mol}}^{-1}$	$\Delta G_{\mathrm{form}}$/kcal·${\mathrm{mol}}^{-1}$
Ethane	−92.3	−81.42
HPE	−11.4	6.9
HPEMe	−15.9	2.6
HPEEt	−23.3	−3.0
HPEiPr	−31.4	−11.7
HPEtBut	−41.3	−18.7

**Table 2:** Delocalization indices and selected topological properties of the electron density in atomic units at the bond critical points of the central C–C bonds in ethane and the compounds schematized in Figure 2A. The corresponding computed bond lengths (angstroms) and formation energies (kcal/mol) for the formation of these chemical bonds are reported as well.

Molecule	δ(C,C)	ρ(r)	$\nabla^{2}\rho \left(r\right)$	H(r)	d(C,C)	$\Delta E_{\mathrm{form}}$
H_3_C–CH_3_	1.006	0.244	−0.5597	−0.196	1.519	−99.7
HPE	0.853	0.182	−0.3325	−0.121	1.685	−14.9
HPEMe	0.841	0.176	−0.3130	−0.115	1.703	−20.4
HPEEt	0.860	0.186	−0.3451	−0.125	1.674	−26.9
HPEiPr	0.860	0.188	−0.3531	−0.128	1.667	−36.5
HPEtBut	0.865	0.188	−0.3528	−0.127	1.669	−45.6

**Table 3:** IQA interaction and deformation energies (Equation (26)) for the formation of ethane and hexaphenylethane from the corresponding free radicals. The values are reported in kcal/mol. The IQA interaction energy for the central C–C bond is written in parentheses. The entry IQA-D stands for the sum of the IQA interaction energy and the contribution of the dispersion correction.

Ethane			
	IQA partition	Dispersion correction	IQA-D
Interaction energy	−196.3 (−172.7)	−0.9	−197.2
Deformation energy	97.2	0.1	−97.3
Formation energy	−99.0	−0.8	−99.9
Hexaphenylethane			
	IQA partition	Dispersion correction	IQA-D
Interaction energy	−291.2 (−147.7)	−20.2	−311.4
Deformation energy	300.9	−0.7	300.2
Formation energy	9.6	−20.9	−11.2

**Table 4:** Collective 1–3 interactions schematized in Figure 2B within the compounds of Figure 2 computed as approximated IQA interaction energies. The magnitude of these interactions are calculated with Equations (16)–(18) and (23). The dispersion component is also reported. The quantity IQA-D indicates the sum of the IQA interaction energy and the value corresponding to the dispersion correction. The values are reported in kcal/mol.

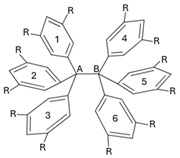			
HPE			
Groups	IQA partition	Dispersion correction	IQA-D
A–4	−3.4	−0.6	−3.9
A–5	−3.4	−0.6	−3.9
A–6	−3.4	−0.6	−4.0
B–1	−3.4	−0.6	−4.0
B–2	−3.4	−0.5	−3.9
B–3	−3.4	−0.6	−4.0
Total	−20.4	−3.3	−23.7
HPEMe			
Groups	IQA partition	Dispersion correction	IQA-D
A–4	−3.4	−0.6	−4.0
A–5	−3.4	−0.6	−4.0
A–6	−3.5	−0.6	−4.1
B–1	−3.5	−0.6	−4.0
B–2	−3.4	−0.6	−4.0
B–3	−3.5	−0.6	−4.1
Total	−20.6	−3.5	−24.1
HPEEt			
Groups	IQA partition	Dispersion correction	IQA-D
A–4	−3.4	−0.6	−4.0
A–5	−3.4	−0.6	−4.0
A–6	−3.5	−0.6	−4.1
B–1	−3.5	−0.6	−4.1
B–2	−3.4	−0.6	−4.0
B–3	−3.5	−0.6	−4.1
Total	−20.7	−3.6	−24.2
HPEiPr			
Groups	IQA partition	Dispersion correction	IQA-D
A–4	−3.3	−0.6	−4.0
A–5	−3.5	−0.6	−4.1
A–6	−3.6	−0.6	−4.2
B–1	−3.4	−0.6	−4.1
B–2	−3.5	−0.6	−4.1
B–3	−3.4	−0.6	−4.0
Total	−20.7	−3.7	−24.4
HPEtBut			
Groups	IQA partition	Dispersion correction	IQA-D
A–4	−3.4	−0.6	−4.1
A–5	−3.3	−0.6	−3.9
A–6	−3.5	−0.6	−4.1
B–1	−3.4	−0.6	−4.1
B–2	−3.3	−0.6	−3.9
B–3	−3.5	−0.6	−4.1
Total	−20.4	−3.8	−24.2

**Table 5:** IQA interaction energies (Equation (23)) among the phenyl groups bonded to the central carbon atoms of hexaphenylethane. The dispersion component is also reported. The numbering of the phenyl groups is indicated in the scheme. The quantity IQA-D denotes the sum of the component of the IQA electronic energy partition and the contribution of the dispersion correction. The values are reported in kcal/mol.

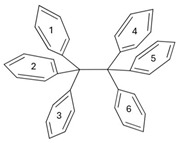			
Phenyl Groups	IQA Partition	Dispersion Correction	IQA-D
1–4	−28.2	−3.6	−31.8
1–5	−10.3	−1.4	−11.7
1–6	−0.2	−0.6	−0.8
2–4	−0.2	−0.6	−0.8
2–5	−28.2	−3.6	−31.8
2–6	−10.3	−1.4	−11.7
3–4	−10.3	−1.4	−11.6
3–5	−0.2	−0.6	−0.8
3–6	−28.0	−3.6	−31.7
Total	−115.9	−16.7	−132.7

**Table 6:** Approximate IQA interaction energies (Equations (16)–(18) and (23)) among the phenyl groups bonded to the central carbon atoms of the molecules shown in Figure 2. The approximate IQA interaction energy for the central C–C is indicated in the last row for every system. The LD component is also reported. The numbering of the phenyl groups is indicated in the scheme. The entry IQA-D denotes the sum of the IQA interaction energy and the contribution of the dispersion correction. The values are reported in kcal/mol.

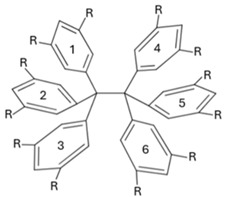			
HPE			
Phenyl groups	IQA partition	Dispersion Correction	IQA-D
1–4	−15.6	−3.586	−19.194
1–5	−7.2	−1.4	−8.6
1–6	−0.5	−0.6	−1.1
2–4	−0.5	−0.6	−1.1
2–5	−15.6	−3.6	−19.2
2–6	−7.2	−1.4	−8.6
3–4	−7.2	−1.4	−8.6
3–5	−0.5	−0.6	−1.1
3–6	−15.5	−3.6	−19.1
Total	−69.9	−16.7	−86.6
C–C	−83.9		
HPEMe			
Phenyl groups	IQA partition	Dispersion correction	IQA-D
1–4	−18.3	−5.4	−23.7
1–5	−7.4	−1.6	−9.0
1–6	−0.5	−0.7	−1.2
2–4	−0.5	−0.7	−1.2
2–5	−18.3	−5.5	−23.8
2–6	−7.1	−1.6	−8.7
3–4	−7.1	−1.6	−8.6
3–5	−0.5	−0.7	−1.2
3–6	−18.3	−5.2	−23.5
Total	−78.0	−23.0	−101.0
C–C	−81.9		
HPEEt			
Phenyl groups	IQA partition	Dispersion correction	IQA-D
1–4	−24.3	−6.9	−31.3
1–5	−8.4	−2.0	−10.4
1–6	−0.5	−0.7	−1.2
2–4	−0.5	−0.7	−1.3
2–5	−25.8	−7.6	−33.4
2–6	−8.3	−2.0	−10.3
3–4	−9.3	−2.3	−11.5
3–5	−0.5	−0.7	−1.2
3–6	−21.2	−6.3	−27.6
Total	−99.0	−29.1	−128.1
C–C	−85.2		
HPEiPr			
Phenyl groups	IQA partition	Dispersion correction	IQA-D
1–4	−20.3	−6.3	−26.7
1–5	−19.3	−5.6	−24.9
1–6	−0.7	−0.7	−1.4
2–4	−0.7	−0.7	−1.3
2–5	−15.8	−4.7	−20.5
2–6	−15.3	−4.8	−20.1
3–4	−16.9	−5.1	−22.0
3–5	−0.6	−0.6	−1.3
3–6	−17.4	−5.3	−22.7
Total	−107.0	−33.8	−140.8
C–C	−85.1		
HPEtBut			
1–4	−30.7	−9.6	−40.3
1–5	−12.9	−3.6	−16.5
1–6	−0.5	−0.9	−1.4
2–4	−0.5	−0.9	−1.4
2–5	−30.8	−9.6	−40.3
2–6	−11.4	−3.5	−14.9
3–4	−12.9	−3.6	−16.5
3–5	−0.5	−0.9	−1.4
3–6	−29.6	−9.7	−39.2
Total	−129.8	−42.1	−171.9
C–C	−86.0		

**Table 7:** Delocalization indices among the aromatic groups bonded to the central carbon atoms of the molecules shown in Figure 2A. The delocalization indices for the central C–C bonds are reported at the bottom of the chart. Atomic units are used throughout.

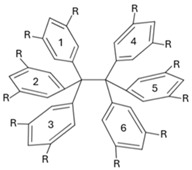					
Phenyl Groups	HPE	HPEMe	HPEEt	HPEiPr	HPEtBut
1–4	0.298	0.364	0.473	0.377	0.593
1–5	0.111	0.112	0.129	0.354	0.216
1–6	0.016	0.017	0.017	0.022	0.017
2–4	0.016	0.017	0.017	0.021	0.017
2–5	0.300	0.367	0.504	0.281	0.594
2–6	0.111	0.111	0.129	0.270	0.194
3–4	0.111	0.110	0.149	0.308	0.216
3–5	0.016	0.017	0.016	0.020	0.017
3–6	0.299	0.358	0.414	0.313	0.574
Total	1.278	1.471	1.847	1.966	2.438
C–C	0.853	0.841	0.860	0.861	0.865

**Table 8:** Index $\Theta_{\mathrm{col}/\mathrm{cov}}$ as computed with Equation (2) for the molecules examined throughout this investigation. The collective 1–3 and 1–4 interactions are schematized in Figure 2B.

Molecule	$\Theta_{\mathrm{col}/\mathrm{cov}}$
Ethane	0.141
HPE	1.108
HPEMe	1.552
HPEEt	1.803
HPEiPr	1.915
HPEtBut	2.288

## Data Availability

The original contributions presented in this study are included in the article and Supplementary Material. Further inquiries can be directed to the corresponding authors.

## Supplementary Materials

The following supporting information can be downloaded at: https://www.mdpi.com/article/10.3390/molecules30214316/s1, Table S1: Computed changes in energy for the process $2{\left(\mathrm{Ph}\right)}_{3}C\cdot ⇋{\left(\mathrm{Ph}\right)}_{3}{C\text{-}C(\mathrm{Ph})}_{3}$, with different exchange correlation functionals and basis sets.
