# Supplementary material for: Interacting Quantum Atoms Analysis of Covalent and Collective Interactions in Single Elongated Carbon–Carbon Bonds
Source: Molecules. 2025 Nov 6;30(21):4316. doi: 10.3390/molecules30214316 (PMC12609698; doi:10.3390/molecules30214316)
Supplement: Supplementary file 1 [file molecules-30-04316-s001.zip › molecules-3925400-supplementary.pdf]

Interacting Quantum Atoms Analysis of Covalent and Collective Interactions in Single Elongated Carbon–Carbon Bonds

Antonio Bonesana-Espinoza [1], José Manuel Guevara-Vela [2], Evelio Francisco [3], Tomás Rocha-Rinza [1,*] and Ángel Martín Pendás [3,**]

[1]*Instituto de Química, Universidad Nacional Autónoma de México, Circuito Exterior, Ciudad Universitaria, Delegación Coyoacán C.P. 04510, Mexico City, Mexico.*

[2]*School of Engineering and Physical Sciences, Heriot-Watt University, Edinburgh EH14 4AS, UK*

[3]*Departamento de Química Física y Analítica, Universidad de Oviedo, Av. Julián Clavería 8, 33006 Oviedo, Asturias, Spain*

XYZ files

In this link

https://doi.org/10.5281/zenodo.17482779

the interested reader can download the XYZ files and AIMAll outputs (in sumviz format) corresponding to the molecules addressed in this investigation as well as the Python scripts used to compute the LHS of equations (15) and (16), i.e., the approximations to $E_{\text{cl}}^{\text{AB}}$ and $E_{\text{xc}}^{\text{AB}}$ respectively, along with the dispersion contributions to the IQA interaction energies.

*To whom correspondence should be addressed: trocha@iquimica.unam.mx

**To whom correspondence should be addressed: ampendas@uniovi.es

Benchmark of exchange-correlation functionals

Table S1: Computed changes in energy for the process $(Ph)_3C\cdot \leftrightharpoons (Ph)_3C{-}C(Ph)_3$ with different exchange-correlation functionals and basis sets.

PBE/Def2-SVP	8.80
PBE/Def2-TZVP	14.10
PBE+D3BJ/Def2-SVP	−12.00
PBE+D3BJ/Def2-TZVP	−6.80
PBEh-3c	−6.10
PBE0/Def2-SVP	5.70
PBE0/Def2-TZVP	11.10
M06-2X/Def2-SVP	−16.60
M06-2X/Def2-TZVP	−11.10
PBE0+D3BJ/Def2-SVP	−14.70
PBE0+D3BJ/Def2-TZVP	−9.40
PBE0+D3BJ/aug-cc-PVTZ	0.00
B3LYP/Def2-SVP	20.60
B3LYP/Def2-TZVP	26.40
B3LYP +D3BJ/Def2-SVP	−11.50
B3LYP +D3BJ/Def2-TZVP	−5.70
DLPNO-CCSD(T)/cc-pVTZ	−22.40
